# circACTA2 mediates Ang II-induced VSMC senescence by modulation of the interaction of ILF3 with CDK4 mRNA

**DOI:** 10.18632/aging.202855

**Published:** 2021-04-22

**Authors:** Ying Ma, Bin Zheng, Xin-Hua Zhang, Zi-Yuan Nie, Jing Yu, Hong Zhang, Dan-Dan Wang, Bei Shi, Yang Bai, Zhan Yang, Jin-Kun Wen

**Affiliations:** 1 Department of Biochemistry and Molecular Biology, Key Laboratory of Neural and Vascular Biology, Ministry of Education, Hebei Medical University, Shijiazhuang 050017, China; 2 Department of Urology, The Second Hospital of Hebei Medical University, Shijiazhuang 050000, China; 3 Department of Biochemistry and Molecular Biology, Binzhou Medical University, Yantai 264003, China

**Keywords:** senescence, VSMC, circACTA2, ILF3, CDK4

## Abstract

Chronic angiotensin II (Ang II) stimulation induces vascular smooth muscle cell (VSMC) senescence, and circRNAs and members of the ILF3 family are implicated in cellular senescence, but the mechanism underlying regulation of circRNAs and ILF3 by Ang II in VSMCs remains poorly understood. Here, a model of Ang II-induced VSMC senescence and the renal artery of hypertensive patients were used to investigate the roles and mechanisms of circACTA2 and ILF3 in VSMC senescence. We show that circACTA2 expression was elevated in Ang II-stimulated VSMCs and in the vascular walls of hypertensive patients. circACTA2 knockdown largely abrogated Ang II-induced VSMC senescence as shown by decreased p21 expression and increased CDK4 expression as well as by decreased SA β-gal-positive cells. Oligo pull-down and RIP assays revealed that both circACTA2 and CDK4 mRNA could bind with ILF3, and Ang II facilitated circACTA2 association with ILF3 and attenuated ILF3 interaction with CDK4 mRNA. Mechanistically, increased circACTA2 by Ang II reduced ILF3 association with CDK4 mRNA by competing with CDK4 mRNA to bind to ILF3, which decreases CDK4 mRNA stability and protein expression, thus leading to Ang II-induced VSMC senescence. Targeting the circACTA2-ILF3-CDK4 axis may provide a novel therapeutic strategy for VSMC senescence-associated cardiovascular diseases.

## INTRODUCTION

One of the hallmarks of aging is the accumulation of senescent cells in aged organisms, thereby leading to a progressive loss of tissue and organ functions and high risk of age-related diseases [1, 2]. Among all age-related diseases, cerebra-cardiovascular diseases are the leading cause of mortality [1]. Emerging evidence has shown that senescent cells accumulate with age in multiple tissues, including arterial walls [3]. Vascular smooth muscle cells (VSMC) are the sole cellular components in the arterial medial layer and play critical roles in vascular structure and function. The senescent VSMCs acquire the senescence-associated secretory phenotype (SASP), which is relevant for pro-inflammatory cytokine secretion and chronic vascular inflammation [4]. Senescent cells also exhibit increased gene expression of p53, p21 and p16, which are negative regulators of the cell cycle, as well as increased activity of senescence-associated β-galactosidase (SA-β-gal), a biomarker of cellular senescence [5, 6]. The senescence of VSMCs has been demonstrated to be closely associated with age-related vascular disorders such as hypertension, atherosclerosis and aneurysm [1, 7, 8]. Although VSMC senescence may be provoked by many factors, such as inflammation, oxidative stress, DNA damage, etc, angiotensin II (Ang II) plays critical roles in the pathogenesis of VSMC senescence by triggering inflammation and oxidative stress, which are initiated by activating nuclear transcription factor-kappa B (NF-κB) through the AT1 and AT2 receptors, as well as by upregulating the expression of MMP-2/-9, calpain-1, and TGF-β1 [7]. Despite the emerging evidence showing that SIRT1 (Sirtuin 1) reduction in VSMCs facilitated Ang II-induced vascular aging and aneurysm formation [9] and the accumulation of SM22α promoted Ang II-induced VSMC senescence through suppressing Mdm2-mediated ubiquitination and degradation of p53 [10], the molecular mechanisms whereby chronic Ang II stimulation induces VSMC senescence remain incompletely understood.

Circular RNAs (circRNAs) are a new class of noncoding RNAs characterized by covalently closed loop structures. The circular structure, which is formed by the backsplicing, makes circRNAs more stable than linear mRNAs [11]. Accumulating evidence suggests that circRNAs exist specifically in different cells and tissues and play key roles in various cellular processes as well as in many pathophysiological mechanisms [12, 13]. circRNAs are mainly distributed in the cytoplasm, where they function as miRNA sponges, protein sponges, or scaffolds for the formation of protein complexes [14]. In recent years, several lines of evidence have shown that circRNAs play an important role in the regulation of autophagy, apoptosis, necrosis, senescence and inflammation in cardiovascular cells [15] and are implicated in cardiovascular diseases [16]. For example, circ-Foxo3 promotes cardiac senescence by interacting with the anti-senescent protein ID-1 and the transcription factor E2F1, as well as the anti-stress proteins FAK and HIF1α [17]. circ-SATB2 regulates VSMC proliferation, differentiation, apoptosis and migration by promoting the expression of STIM1 [18]. Circ_Lrp6 acts as a sponge regulating miRNA-145 function to hinder miR-145-mediated regulation of VSMC migration, proliferation and differentiation [19]. Our previous work showed that circACTA2 mediates NRG-1-ICD regulation of α-SMA expression in VSMCs via binding to miR-548f-5p [20]. However, the roles of circACTA2 in VSMC senescence and the underlying mechanisms are currently unknown.

Interleukin enhancer-binding factor 3 (ILF3) and nuclear factor 90 (NF90) are two ubiquitously expressed proteins generated by alternative splicing of the Ilf3 gene [21]. Since they contain double-stranded RNA (dsRNA)-binding motifs which interact with AU-rich elements (ARE) present in the 3′ untranslated region (UTR) of various RNAs, ILF3 and NF90 can bind to the 3′ UTR of several mRNAs to regulate their stability, leading to mRNA degradation or stabilization in a context-dependent manner [22]. For instance, NF90 (ILF3) was found to bind to the 3' UTR of cyclin E1 mRNA *in vitro* and *in vivo*, and overexpression of NF90 increases cyclin E1 protein expression by stabilizing cyclin E1 mRNA [23]. In contrast, ILF3 was reported to bind to BAFF WT-mRNA in human monocytic leukemia THP-1 cells, suppressing BAFF translation by recruiting miR-15a to the 3' UTR of BAFF-WT mRNA [24]. LincIN, a NF90-binding long non-coding RNA, binds to ILF3 and together inhibit p21 expression at the translational level [25]. Additionally, ILF3/NF90 promotes circRNA biogenesis and interacts with circRNAs in the cytoplasm. Upon viral infection, the de-association of ILF3/NF90 from circRNP complexes allows their binding to viral mRNAs to inhibit viral replication [26]. Despite the accumulating evidence suggesting that members of the ILF3 family are involved in all steps of RNA metabolism, as well as in the numerous and diverse functions, the relationship among circACTA2, ILF3 and Ang II-induced VSMC senescence has not yet been elucidated.

In the present study, our findings showed that expression of circACTA2 is significantly upregulated in VSMCs chronically stimulated with Ang II. Increased circACTA2 reduces the association of ILF3 and CDK4 mRNA by competing with CDK4 mRNA to bind to ILF3, which decreases CDK4 mRNA stability and protein expression, thus leading to Ang II-induced VSMC senescence. Targeting the circACTA2-ILF3-CDK4 may provide a novel therapeutic strategy for VSMC senescence-associated cardiovascular diseases

## RESULTS

### circACTA2 expression is upregulated in Ang II-induced VSMC senescence and in artery tissues of hypertensive patients

**Figure 1:**
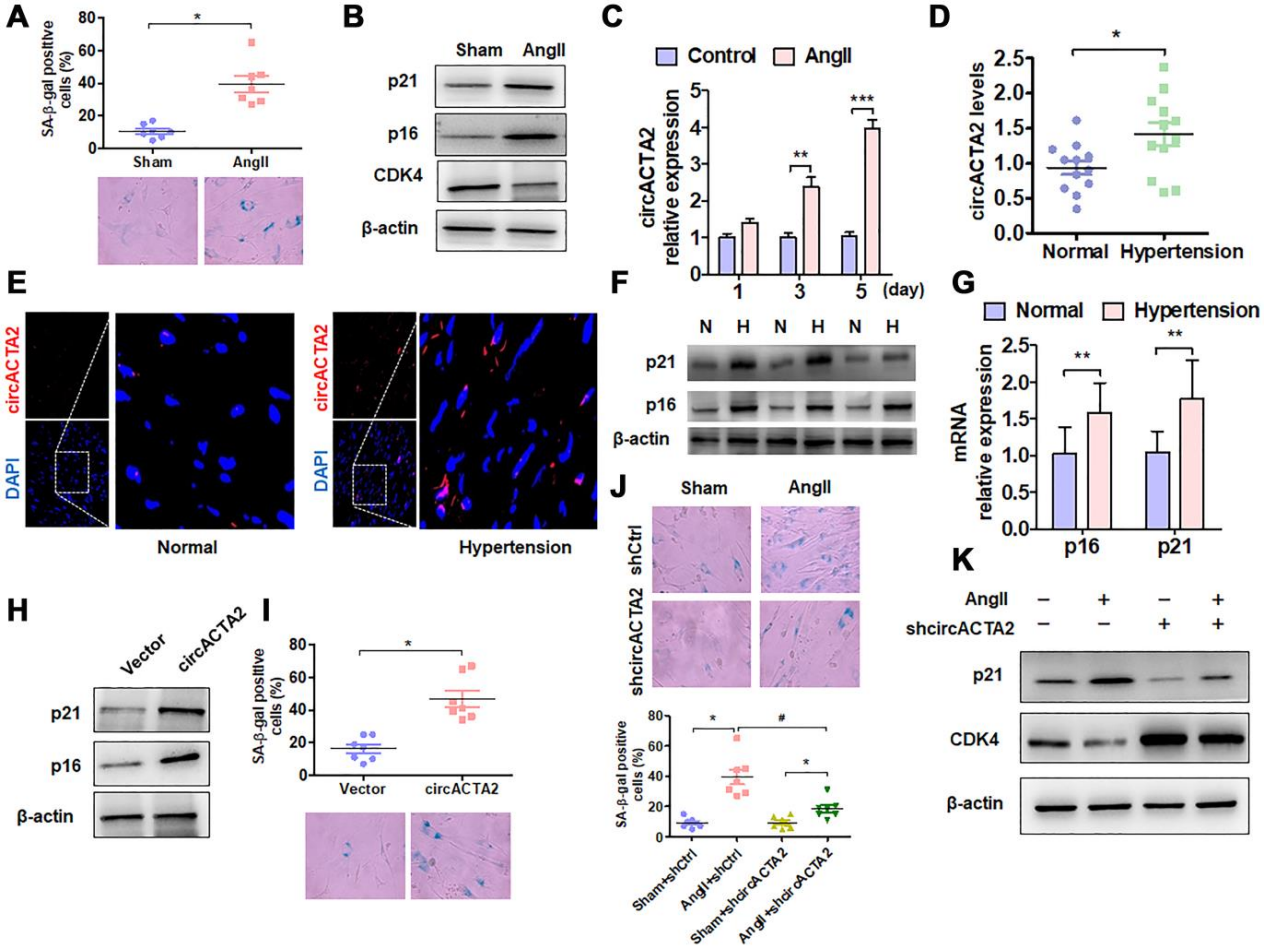
**circACTA2 is upregulated in Ang II-induced VSMCs and in the vascular walls of old mice.** (**A**) SA-β-gal activity in VSMCs treated with or without Ang II (100 nmol/L) for 3 days. The percentage of SA-β-gal positive cells (above) and representative pictures (below) are shown. Magnification ×400. ^*^P < 0.05 vs. vehicle control. (**B**) Western blot detection of p21, p16 and CDK4 expression in VSMCs treated with or without Ang II (100 nmol/L) for 3 days. (**C**) VSMCs were serum-starved for 24 h and then treated with or without Ang II (100 nmol/L) for 1, 3 and 5 days. circACTA2 expression was measured by qRT-PCR analysis. Data represent the means ± SEM of 3 independent experiments. ^**^P < 0.01, ^***^P < 0.001 vs. vehicle control. (**D**) qRT-PCR detection of circACTA2 expression in the renal artery of hypertensive patients and matched controls. ^*^P < 0.05 vs. control. (**E**) *In situ* hybridization of circACTA2 (red) in the renal artery of hypertensive patients and controls. (**F**) Western blot detected p21 and p16 expression in artery tissues of normal controls (N) and hypertensive patients (**H**). (**G**) qRT-PCR detected p21 and p16 mRNA expression in artery tissues of hypertensive patients and normal controls. ^**^P < 0.01 vs. control. (H) Western blot detection of p21 and p16 expression in VSMCs transfected with empty vector or circACTA2. (**I**) SA-β-gal activity in VSMCs transfected with empty vector or circACTA2. The percentage of SA-β-gal positive cells (above) and representative pictures (below) are shown. Magnification × 400. ^*^P < 0.05 vs. empty vector. (**J**) SA-β-gal activity in VSMCs transfected with shCtrl or shcircACTA2 followed by treatment with or without Ang II. The percentage of SA-β-gal positive cells (left) and representative pictures (right) are shown. Magnification × 400. ^*^P < 0.05, ^#^*P* < 0.05 vs. their corresponding control. (**K**) Western blot detection of p21 and CDK4 expression in VSMCs transfected with shCtrl or shcircACTA2 followed by treatment with or without Ang II.

To evaluate the importance of circACTA2 in cellular senescence of VSMCs, we first established a model of VSMC senescence induced by Ang II that has been proven to be involved in VSMC senescence and vascular aging in cell or animal models [1, 7]. The characteristic of VSMC senescence was evaluated by using β-galactosidase (SA β-gal) staining, a classical method for detecting cellular senescence. When VSMCs were exposed to Ang II (100 nmol/L) or vehicle for 3 days, the number of SA β-gal-positive cells was significantly increased relative to vehicle control (Figure 1A). Also, we used western blot analysis to detect the expression of senescence-associated genes p21 and p16 as well as positive cell-cycle regulator CDK4 in Ang II-treated VSMCs. The results showed that the expression level of p21 and p16 was significantly upregulated by chronic Ang II stimulation, whereas CDK4 expression was decreased under the same conditions compared with vehicle control, suggesting that chronic Ang II stimulation has resulted in VSMC senescence (Figure 1B). Based on our previous observations that circACTA2 could promote the expression of smooth muscle α-actin (α-SMA) that is implicated in cellular contraction and senescence [20], we hypothesized that circACTA2 might participate in Ang II-induced cellular senescence in VSMCs. To test this, we examined whether Ang II affects circACTA2 expression and cellular proliferation. As shown in Figure 1C, circACTA2 expression was significantly upregulated in VSMCs exposed to Ang II for 3 or 5 days relative to vehicle control, with a depressed cellular proliferation (Supplementary Figure 1). These results indicated that Ang II induces VSMC senescence and that circACTA2 upregulation may be correlated with Ang II-induced VSMC senescence.

To determine the pathophysiological relevance of circACTA2 expression, we detected the expression level of circACTA2 in artery tissues of patients with hypertension, an age-related vascular disease, by RT-qPCR and FISH. The results showed that a higher level of circACTA2 expression was observed in the artery tissues of hypertensive patients than in the normal vessel tissues (Figure 1D). FISH results showed that circACTA2 was localized in the cytoplasm and upregulated in the vessel tissues of hypertensive patients (Figure 1E). To determine whether the expression of senescence-associated genes was altered in artery tissues of patients with hypertension, we performed RT-qPCR and Western blot to detect p16 and p21 expression in vascular tissues. The results showed that p16 and p21 protein and mRNA levels were significantly upregulated in artery tissues of hypertensive patients (Figure 1F and 1G). Next, we explored whether the upregulation of circACTA2 is responsible for VSMC senescence. We overexpressed circACTA2 in VSMCs by transfecting circACTA2 expression plasmids (Supplementary Figure 2A). We found that the expression level of p21 and p16 was substantially elevated in circACTA2-overexpressing cells (Figure 1H), accompanied by a corresponding increase in the number of SA β-gal-positive cells (Figure 1I). These results imply that circACTA2 plays an important role in VSMC senescence. To further test whether Ang II-induced VSMC senescence is mediated by circACTA2, circACTA2 was knocked down in VSMCs by transfection of plasmids expressing circACTA2 short hairpin RNA (shcircACTA2) (Supplementary Figure 2B), and Ang II-induced VSMC senescence was examined. As a result, knockdown of circACTA2 significantly reduced VSMC senescence induced by Ang II, as shown by decreased number of SA β-gal-positive cells (Figure 1J and Supplementary Figure 2C). Consistently, Ang II-induced upregulation of p21 expression level was abrogated in circACTA2-knocked down VSMCs (Figure 1K), and the knockdown of circACTA2 reversed the inhibitory effect of Ang II on CDK4 expression (Figure 1K). These findings indicate that Ang II induces cellular senescence at least in part by upregulating circACTA2 in VSMCs.

**Figure 2:**
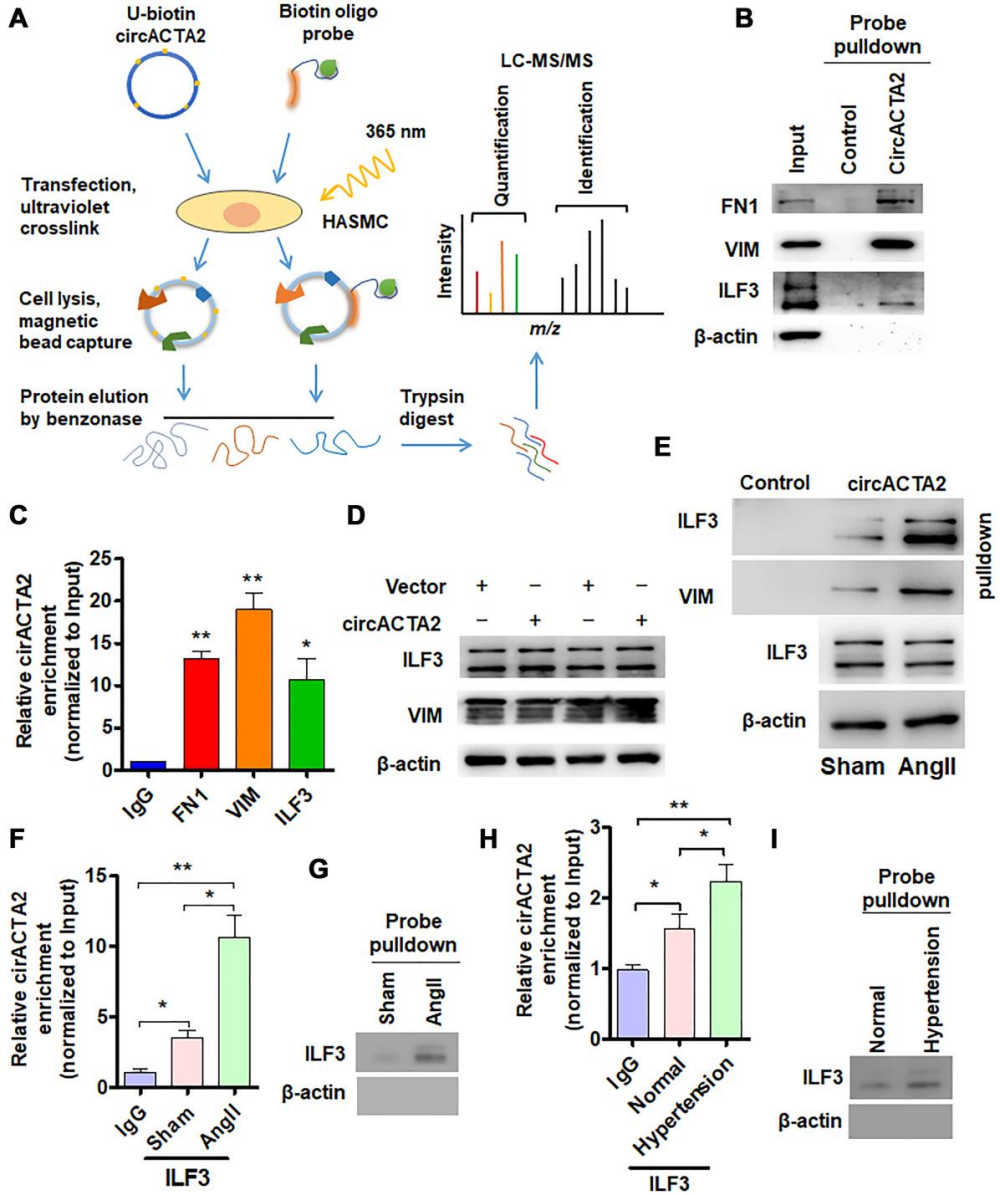
**circACTA2 interacts with ILF3 in VSMCs.** (**A**) Schematic of the experimental design based on RNA antisense purification (RAP) or U-biotin-labeled RNA pull-down followed by LC-MS/MS analysis. (**B**) Western blot detection of FN1, VIM and ILF3 in the circACTA2 probe-pulled down precipitates (circACTA2-overexpressed group vs. control group). (**C**) qRT-PCR detection of circACTA2 level in the RNA-protein immunoprecipitates pulled down by anti-FN1, anti-VIM and anti-ILF3 antibodies. ^*^P < 0.05, ^**^P < 0.01 vs. IgG. (**D**) Western blot detection of ILF3 and VIM expression in VSMCs transfected with empty vector or circACTA2. (**E**) VSMCs were transfected with circACTA2 and treated with or without Ang II, and then cell lysates were pulled down with circACTA2 probe and detected by western blotting with anti-ILF3 and anti-VIM antibodies. (**F**, **H**) RIP-PCR detected the circACTA2 and ILF3 interaction in VSMCs treated with or without Ang II (F) or in artery tissues of hypertensive patients (H). ^*^P < 0.05, ^**^P < 0.01 vs. their corresponding control. (**G**, **I**) Probe of circACTA2 was used to detect ILF3 and circACTA2 interaction in VSMCs treated with or without Ang II (G) or in artery tissues of hypertensive patients (I).

### circACTA2 interacts with ILF3 in VSMCs

In order to elucidate how circACTA2 acts as a regulator of cellular senescence, we identified proteins interacting with circACTA2 by using both RNA antisense purification (RAP) and U-biotin-labeled circACTA2 pull-down followed by quantitative liquid chromatography-mass spectrometry (LC-MS) (Figure 2A). We quantitatively compared the proteins captured with antisense oligonucleotides or biotinylated circACTA2 with their corresponding control-captured proteins. As a result, 181 proteins were quantified. Among these proteins, each had more than two unique peptides in the two biological replicate purifications performed, indicating the suitability of the approach. We reproducibly identified the part of circACTA2-strongly enriched proteins that met our enrichment criteria (mean log2 > 2.0, *P* < 0.05, moderated *t*-test), and confirmed that circACTA2 interaction with FN1, VIM and ILF3, but not β-actin, was highly specific, as detected by immunoblotting with anti-FN1, anti-VIM and anti-ILF3 antibodies (Figure 2B). RNA immunoprecipitation (RIP) performed with these three antibodies further validated their interaction (Figure 2C). Among these, ILF3, a RNA-binding protein, has attracted our attention, which binds to the 3′ untranslated region (UTR) of several mRNAs to enhance mRNA stability and is implicated in regulation of the cell cycle [21]. Therefore, we further investigated whether circACTA2 affects ILF3 expression, and whether Ang II impacts the interaction between circACTA2 and ILF3. Western blot analysis showed that overexpression of circACTA2 in VSMCs, which mimicked Ang II stimulation, did not significantly affect ILF3 and VIM expression (Figure 2D). However, oligonucleotide pull-down assay performed with the probe of circACTA2 revealed that circACTA2 interaction with ILF3 or VIM was enhanced in Ang II-treated VSMCs (Figure 2E). To examine whether endogenous ILF3 interacts with circACTA2 under pathophysiological condition in VSMCs, we performed the RIP and RNA pulldown. As shown in Figure 2F and 2G, circACTA2 was significantly enriched in the anti-ILF3 immunoprecipitates relative to IgG, and this could be further enhanced in VSMCs treated with Ang II. RNA oligo pulldown also confirmed that Ang II treatment promoted circACTA2 and ILF3 interaction. In the artery tissues, we also found that compared with normal artery tissues, ILF3 and circACTA2 interaction significantly increased in the artery tissues of hypertensive patients (Figure 2H and 2I). These results suggest that circACTA2 and ILF3 interaction happens under pathophysiological condition in the cell. Taken together, these results support the notion that circACTA2 interacts with ILF3 in VSMCs and that their interaction is strengthened by Ang II exposure, suggesting that circACTA2 interaction with ILF3 might be involved in Ang II-induced cellular senescence of VSMCs.

### ILF3 overexpression suppresses Ang II-induced VSMC senescence

**Figure 3:**
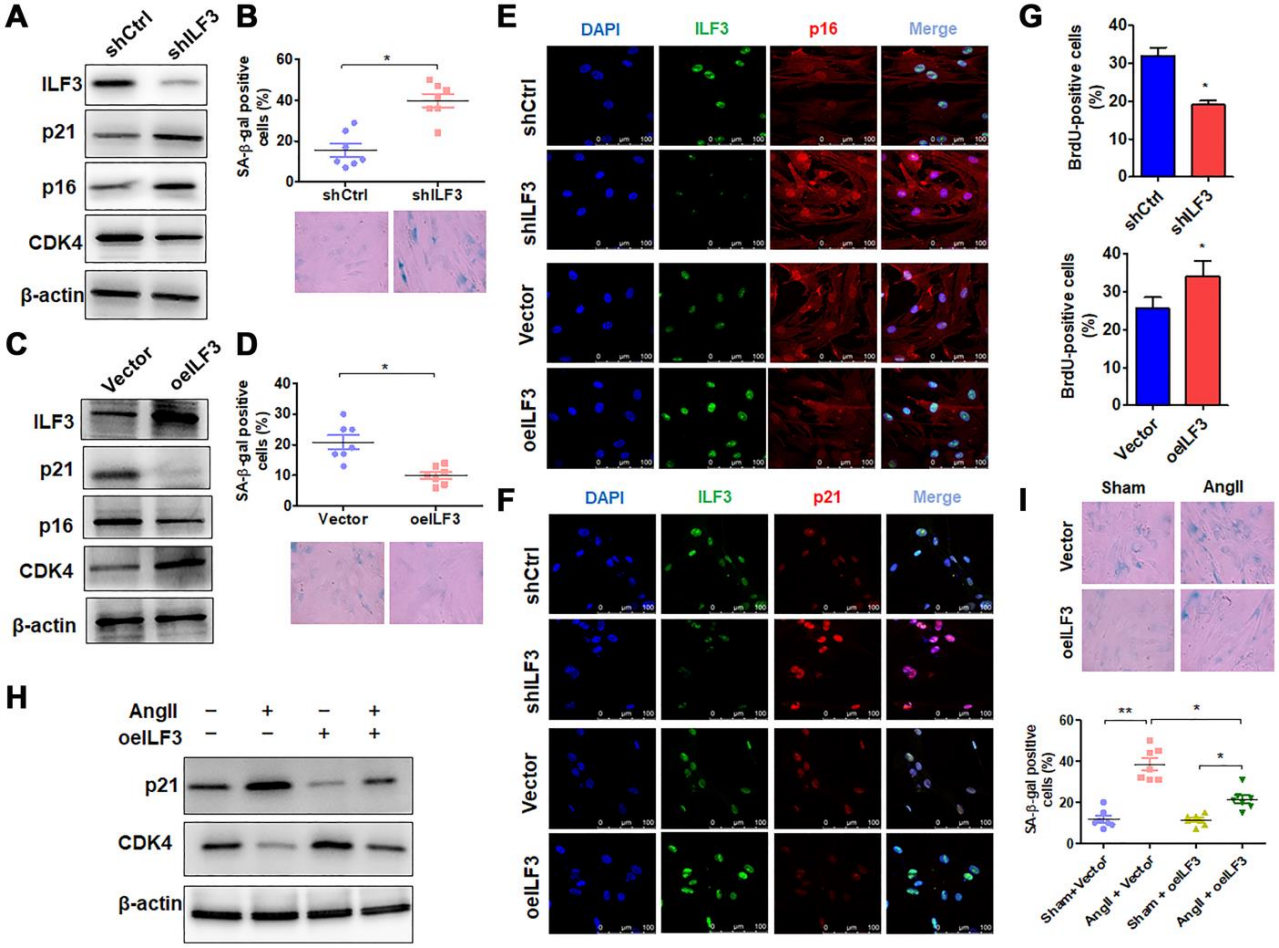
**ILF3 overexpression suppresses Ang II-induced VSMC senescence.** (**A**) Western blot detection of ILF3, p21, p16 and CDK4 expression in VSMCs transfected with shCtrl or shILF3. (**B**) SA-β-gal activity in VSMCs transfected with shCtrl or shILF3. The percentage of SA-β-gal positive cells (above) and representative pictures (below) are shown. Magnification × 400. ^*^P < 0.05 vs. shCtrl. (**C**) Western blot detection of ILF3, p21, p16 and CDK4 expression in VSMCs transfected with empty vector or ILF3-expressing vector (oeILF3). (**D**) SA-β-gal activity in VSMCs transfected with empty vector or oeILF3. The percentage of SA-β-gal positive cells (above) and representative pictures (below) are shown. Magnification ×400. ^*^P < 0.05 vs. empty vector. (**E**) and (**F**) VSMCs were transfected with shILF3 or oeILF3 and their corresponding controls, and the expression of ILF3, p16 and p21 was examined by immunofluorescence staining. Green, red, and blue staining indicates ILF3, p16 (E), p21 (F) and the nuclei, respectively. Scale bar = 100 μm. (**G**) VSMCs were transfected as in (E), and cell proliferation was estimated by BrdU incorporation test. Graph presents means ± SD from at least three independent experiments. ^*^P < 0.05 vs. their corresponding control. (**H**) Western blot detection of p21 and CDK4 expression in VSMCs transfected with empty vector or oeILF3 followed by treatment with or without Ang II. (**I**) SA-β-gal activity in VSMCs transfected with empty vector or oeILF3 followed by treatment with or without Ang II. The percentage of SA-β-gal positive cells (bottom) and representative pictures (top) are shown. Magnification × 400. ^*^P < 0.05, ^**^P < 0.01 vs. their corresponding control.

Because the above findings indicated ILF3 interaction with circACTA2, and previous study has shown that ILF3 functions as a repressor of cellular senescence and its expression is downregulated in senescent fibroblasts [4], we used loss- and gain-of-function approaches to examine the role of ILF3 in VSMC senescence. When VSMCs were transfected with ILF3-specific short hairpin RNA (shILF3) to knock down the expression of ILF3, the expression of cellular senescence markers p21 and p16 was increased in ILF3-silenced VSMCs compared with those transfected with shCtrl, with an obvious decrease in CDK4 expression level, as shown by Western blot analysis (Figure 3A). Accordingly, the proportion of SA β-gal-positive cells was significantly increased in ILF3-knocked down VSMCs (Figure 3B). On the contrary, overexpression of ILF3 mediated by ILF3-expressing vector (oeILF3) reduced p21 and p16 expression and enhanced CDK4 expression level (Figure 3C), accompanied by a marked decrease in the proportion of cells positive for the SA β-gal activity (Figure 3D). Further, immunofluorescence staining of p16 and p21 showed that knockdown of ILF3 increased and overexpression of ILF3 decreased the expression of p16 and p21 compared to their corresponding control (Figure 3E and 3F). To provide additional evidence that ILF3 regulates VSMC senescence, BrdU incorporation assay was performed to evaluate the effect of ILF3 on VSMC proliferation. The results showed that ILF3 silencing by shRNA significantly inhibited the proliferation of VSMCs, whereas its overexpression facilitated VSMC proliferation (Figure 3G). These results clearly indicate that ILF3 suppresses cellular senescence in VSMCs. To further confirm that ILF3 functionally participates in Ang II-induced senescence, we treated ILF3-overexpressing VSMCs with Ang II and assessed cellular senescence via detecting the expression of senescence-associated genes as well as by SA β-gal staining. Western blot analysis showed that overexpression of ILF3 largely abrogated Ang II-induced upregulation of p21 expression and decreased the downregulation of CDK4 expression by Ang II (Figure 3H). Simultaneously, Ang II-induced cellular senescence was rescued by ILF3 overexpression in VSMCs, as evidenced by SA β-gal staining (Figure 3I). These results suggest that ILF3 represses Ang II-induced VSMC senescence via interacting with circACTA2.

### ILF3 inhibits VSMC senescence by binding and stabilizing CDK4 mRNA

**Figure 4:**
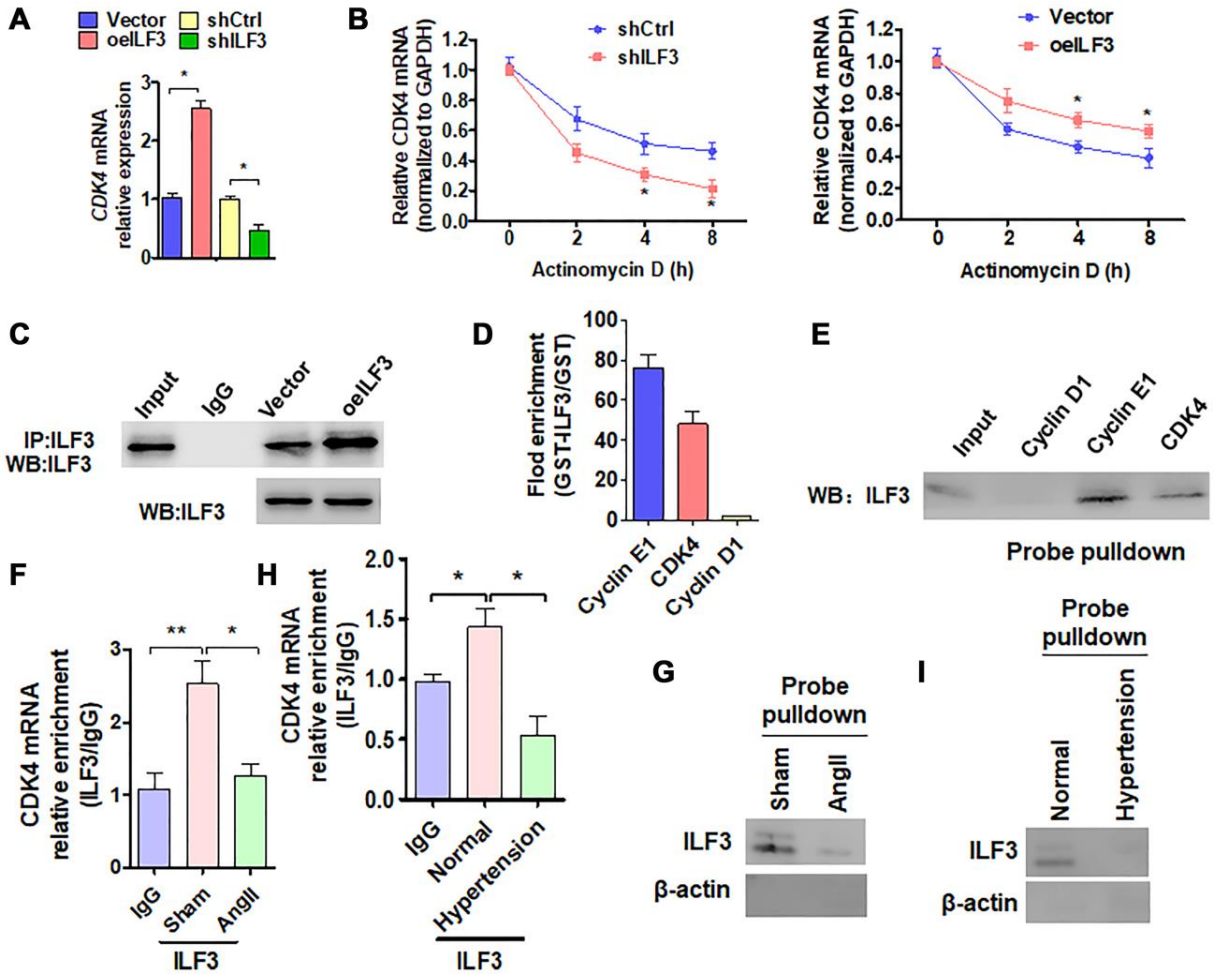
**ILF3 inhibits VSMC senescence by binding and stabilizing CDK4 mRNA.** (**A**) qRT-PCR detection of CDK4 mRNA expression in VSMCs transfected with ILF3-expressing vector (oeILF3) or shILF3 and their corresponding control. Data represent the means ± SEM of 3 independent experiments. ^*^P < 0.05 vs. their corresponding control. (**B**) VSMCs were transfected with shILF3 or oeILF3 and their corresponding control, and then exposed to actinomycin D for 0, 2, 4, and 8 h. CDK4 mRNA level was detected by qRT-PCR. ^*^P < 0.05 vs. shCtrl or empty vector. (**C**) VSMCs were transfected with oeILF3 or empty vector. ILF3 in the anti-ILF3 immunoprecipitates was measured by Western blotting with anti-ILF3 antibody. (**D**) GST pull-down of the indicated mRNAs with recombinant GST-ILF3 or GST from the lysates of VSMCs. The cyclin E1, CDK4 and cyclin D1mRNA on the beads was subjected to qRT-PCR detection. (**E**) The lysates of VSMCs were pulled down with cyclin D1, cyclin E1 or CDK4 3′ UTR probes, and ILF3 in the precipitates was detected by Western blot analysis. (**F**, **H**) RIP-PCR detected the CDK4 mRNA and ILF3 interaction in VSMCs treated with or without Ang II (F) or in artery tissues of hypertensive patients (H). ^*^P < 0.05, ^**^P < 0.01 vs. their corresponding control. (**G**, **I**) Probe of CDK4 mRNA was used to detect ILF3 and CDK4 mRNA interaction in VSMCs treated with or without Ang II (G) or in artery tissues of hypertensive patients (I).

We then investigated the molecular mechanism whereby ILF3 inhibits Ang II-induced VSMC senescence. Based on the above findings that ILF3 overexpression upregulated the expression level of CDK4 which has been known to be negatively correlated with cellular senescence [27], and CDK4/6 inhibitors can induce cellular senescence [28, 29], we hypothesized that ILF3 inhibits VSMC senescence by binding and stabilizing CDK4 mRNA. To test this, we knocked down the expression of CDK4 in VSMCs and found that reduced CDK4 expression could trigger VSMC senescence to a moderate extent (Supplementary Figure 3A and 3B), suggesting that CDK4 can partially counteract VSMC senescence induced by Ang II. ILF3 has been previously shown to associate with numerous SASP **(**senescence-associated secretory phenotype**)** mRNAs to regulate their transcription, translation and mRNA stability [4]. Especially, the findings that ILF3 is able to bind to the 3′ UTR of cyclin E1 mRNA, thus stabilizing cyclin E1 mRNA [23] prompted us to investigate whether ILF3 can also bind to the 3′ UTR of CDK4 mRNA. To test this, ILF3 was knocked down and overexpressed, respectively, in VSMCs and the expression level of CDK4 mRNA was detected by RT-qPCR. As shown in Figure 4A, overexpression of ILF3 increased and knockdown of ILF3 decreased the level of CDK4 mRNA expression compared with their corresponding control. Also, we used RT-qPCR to determine the stability of CDK4 mRNA after treatment of ILF3-silenced or overexpressed VSMCs with actinomycin D. The results showed that knockdown of ILF3 significantly reduced the stability of CDK4 mRNA, while ILF3 overexpression increased it (Figure 4B). These results suggested that ILF3 is required for stabilizing CDK4 mRNA.

We further examined whether ILF3 is bound to the 3′ UTR of CDK4 mRNA. First, CoIP was performed to confirm that enough endogenous ILF3 could be pulled down by anti-ILF3 antibody (Figure 4C). Next, *in vitro* RNA immunoprecipitation (RIP) experiments were performed to examine the association between ILF3 and the CDK4 mRNA 3′ UTR. We expressed and purified recombinant GST-ILF3 fusion protein in Escherichia coli, and used the recombinant protein to pull down mRNAs specifically associated with it. RT-qPCR showed that mRNA of CDK4 and cyclin E1, as a positive control, was dramatically enriched in the recombinant protein-RNA complex pulled down by GST-ILF3. The specificity of ILF3 interaction with cyclin E1 or CDK4 mRNA was confirmed by the absence of association between ILF3 and cyclin D1 mRNA (Figure 4D). Consistent with this, biotin-labeled oligo-nucleotide pull-down followed by western blot analysis also revealed that ILF3 protein could be detected in the precipitates pulled down with the CDK4 3′ UTR probe as well as with cyclin E1 3′ UTR probe, but the cyclin D1 3′ UTR probe could not pull down ILF3 protein (Figure 4E). To further confirm whether endogenous ILF3 protein interacts with CDK4 mRNA under physiological condition, we performed the RIP and RNA pulldown. Compared with the control group, Ang II treatment significantly reduced CDK4 mRNA interaction with ILF3 (Figure 4F and 4G). Similar results were obtained *in vivo*, interaction between CDK4 mRNA and ILF3 was significantly reduced in artery tissues of hypertensive patients relative to the normal artery tissues (Figure 4H and 4I). These findings suggest that endogenous ILF3 protein and CDK4 mRNA interact with each other under pathophysiological condition in VSMCs. These results supported the notion that ILF3 stabilizes CDK4 mRNA by binding to its 3′ UTR.

### circACTA2 promotes VSMC senescence by competing with CDK4 mRNA to bind to ILF3

**Figure 5:**
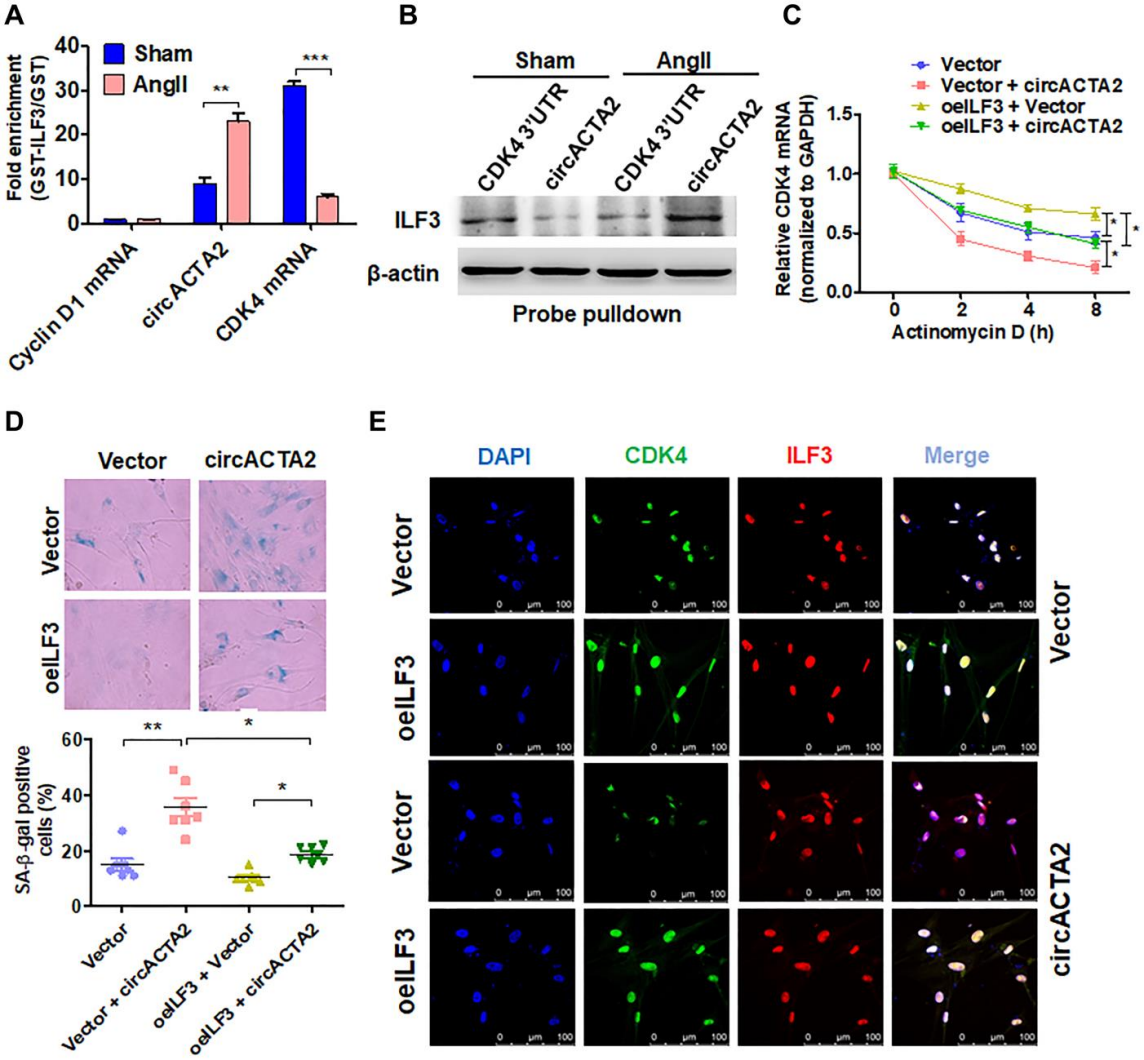
**circACTA2 promotes VSMC senescence by competing with CDK4 mRNA to bind to ILF3.** (**A**) circACTA2, cyclin D1 and CDK4 mRNAs were pulled down with recombinant GST-ILF3 from the lysates of VSMCs treated with or without Ang II for 3 days. The cyclin D1 and CDK4 mRNA as well as circACTA2 on the beads were subjected to qRT-PCR detection. ^**^P < 0.01, ^***^P < 0.001 vs. vehicle control. (**B**) The lysates of VSMCs treated with or without Ang II were pulled down with CDK4 3′ UTR or circACTA2 probe, and ILF3 in the precipitates was detected by Western blot analysis. (**C**) VSMCs were transfected with circACTA2 and ILF3-expressing vector (oeILF3) either alone or together. Then cells were exposed to actinomycin D for 0, 2, 4, and 8 h. CDK4 mRNA level was detected by qRT-PCR. ^*^P < 0.05 vs. their corresponding control. (**D**) SA-β-gal activity in VSMCs transfected as in (C). The percentage of SA-β-gal positive cells (bottom) and representative pictures (top) are shown. Magnification × 400. ^*^P < 0.05, ^**^P < 0.01 vs. their corresponding control. (**E**) VSMCs were transfected as in (C), and the expression of CDK4 and ILF3 was examined by immunofluorescence staining. Green, red, and blue staining indicates CDK4, ILF3, and the nuclei, respectively. Scale bar = 100 μm.

Because both circACTA2 and CDK4 mRNA bind with ILF3, there is a possibility that circACTA2 could compete with CDK4 mRNA for binding to ILF3. To confirm this possibility, interaction of ILF3 with circACTA2 or CDK4 mRNA was examined by RIP experiments after VSMCs were treated with Ang II. The results showed that Ang II treatment caused a significant increase of circACTA2 level in the precipitates pulled down by GST-ILF3, with concomitant reduction of CDK4 mRNA in the precipitates. In contrast, no apparent enrichment was detected for the cyclin D1 mRNA (Figure 5A). To further validate these observations, biotin-labeled oligo-nucleotide pull-down was performed and the ILF3 protein pulled down by circACTA2 probe or the CDK4 3′ UTR probe was determined by immunoblot analysis. As shown in Figure 5B, the association of circACTA2 with ILF3 was enhanced while the interaction of CDK4 mRNA with ILF3 was reduced in Ang II-treated VSMCs compared to Ang II-untreated cells. Further, we performed an *in vivo* competitive binding assay by overexpressing circACTA2 in VSMCs. We co-transfected VSMCs with increasing amounts of circACTA2 expression plasmids and a constant amount of ILF3-expressing vector, and then anti-ILF3 antibody was used to immunoprecipitate RNAs binding to ILF3. As shown in Supplementary Figure 4, when the immunoprecipitate was subjected to RT-qPCR to detect circACTA2 and CDK4 mRNA simultaneously, the level of CDK4 mRNA was gradually decreased with increasing amounts of circACTA2 expression plasmids, accompanied by gradual increase of circACTA2 in the anti-ILF3 immunoprecipitates. These findings suggest that Ang II-increased association of circACTA2 with ILF3 is mainly due to the upregulation of circACTA2 expression by Ang II and then increased circACTA2 competes with CDK4 mRNA for interaction with ILF3, thus attenuating ILF3 interaction with CDK4 mRNA and CDK4 mRNA stability.

Further, we examined the stability of CDK4 mRNA in VSMCs transfected with circACTA2 and ILF3-expressing vector either alone or together and then treated with actinomycin D for the different times. As expected, CDK4 mRNA stability was significantly reduced in circACTA2-overexpressing VSMCs. On the contrary, ILF3 overexpression in VSMCs strengthened the stability of CDK4 mRNA, and increased CDK4 mRNA stability by ILF3 overexpression was abolished by forced co-expression of circACTA2 in VSMCs (Figure 5C). These findings further indicate that circACTA2 upregulation induced by Ang II decreases CDK4 mRNA stability and protein expression via suppressing the association of ILF3 with CDK4 mRNA. Finally, we examined the effect of overexpression of either circACTA2 alone or co-expression of circACTA2 and ILF3 on VSMC senescence. The results showed that overexpression of circACTA2 or ILF3 alone promoted or inhibited, respectively, VSMC senescence. Correspondingly, co-expression of circACTA2 with ILF3 greatly suppressed VSMC senescence elicited by overexpression of circACTA2 (Figure 5D). At the same time, immunofluorescence staining of CDK4 and ILF3 showed that ILF3 overexpression in VSMCs increased the fluorescent intensity of CDK4, and overexpression of circACTA2 attenuated CDK4's fluorescent intensity, but their co-expression largely counteracted the downregulation of CDK4 by overexpression of circACTA2 alone (Figure 5E). Taken together, these findings suggest that circACTA2 mediates Ang II-induced VSMC senescence by competing with CDK4 mRNA to bind to ILF3, thus blocking the association of ILF3 with CDK4 mRNA, which decreases CDK4 mRNA stability and protein expression.

## DISCUSSION

In the present study, we found a novel role of circACTA2 in VSMC senescence induced by Ang II. First, circACTA2 expression was significantly upregulated in Ang II-induced VSMC senescence and in the vascular walls of old mice, and circACTA2 overexpression promoted VSMC senescence, whereas its knockdown had the opposite effects. Second, Ang II-induced VSMC senescence was mediated by circACTA2 interaction with ILF3, which reduces ILF3 association with CDK4 mRNA. Third, decreased interaction of ILF3 with CDK4 mRNA rendered CDK4 mRNA unstable and thus attenuated its protein expression. Fourth, the downregulation of CDK4 expression led to Ang II-induced VSMC senescence.

Emerging evidence has shown that circRNAs are involved in many physiological processes and participate in many pathologic processes including cellular senescence [30]. CircPVT1, one of the senescence-associated circRNAs in human diploid WI-38 fibroblasts, functions as a senescence suppressor by sponging miRNA let-7 and blocking let-7 activity [31]. CircCCNB1 acts as a sponge for miR-449a to delay cellular senescence by targeting cyclin E2 (CCNE2) in human diploid fibroblast 2BS cells [32]. Circ-Foxo3 was reported to have a high expression in heart samples of aged patients and mice [17], and it interacted with the anti-senescence proteins ID1 and E2F1 as well as with anti-stress proteins FAK and HIF1α, kept them in the cytoplasm instead of the nucleus, thus promoting cellular senescence. In the present study, we found that the expression of circACTA2 was significantly elevated in Ang II-induced senescent VSMCs, and overexpression or knockdown of circACTA2 promoted or attenuated VSMC senescence induced by Ang II, respectively. CircRNAs regulate gene expression at transcriptional or post-transcriptional level in eukaryotes by different means [16]. In addition to sponging miRNAs, circRNAs can also bind or sequester specific proteins to modulate their activity or subcellular localization [33, 34]. To dissect the molecular mechanisms underlying circACTA2-mediated Ang II-induced VSMC senescence, we tried to identify circACTA2-associated proteins through RNA pull-down assays followed by quantitative liquid chromatography-mass spectrometry (LC-MS). We confirmed that circACTA2 interacted with ILF3 and that their interaction was highly specific. Furthermore, this interaction was further validated by RNA-binding protein immunoprecipitation (RIP). These findings suggest that circACTA2 interaction with ILF3 might participate in Ang II-induced VSMC senescence.

Regarding the molecular mechanism underlying the regulation of circACTA2 expression by Ang II, it has been widely accepted that RNA-binding proteins (e.g. QKI, also known as Quaking) and RNA-editing enzyme ADAR1 are responsible for the formation of circRNAs. We speculate that both QKI and ADAR1 could participate in Ang II-induced circACTA2 formation in VSMCs. However, how Ang II regulates the QKI and ADAR1 expression needs to be addressed in future studies.

ILF3, also known as NF90 [35], NF110 [36] and TCP80 [37] in humans, is a vital double-stranded RNA-binding protein generated by splicing of the Ilf3 gene [21]. There are a few lines of evidence indicating that ILF3 is associated with cellular senescence. For instance, NF90 has been demonstrated to act as a post-transcriptional repressor of several SASP factors to repress the translation of SASP factors. Senescent human fibroblasts expressed low levels of NF90, thus facilitating cellular senescence [4]. Senescence-associated lncRNA (SALNR) suppressed Ras-induced fibroblast cellular senescence via interacting with NF90 [38]. In general situations, ILF3 inhibits cellular senescence by suppressing the expression of senescence-associated miRNAs, but in the Ras-induced senescent cells, ILF3 translocates to the nucleoli and its inhibitory effect on miRNA biogenesis is eliminated [38]. In this study, an increased expression of p21 and p16 and reduced level of CDK4 were observed when ILF3 was knocked down in VSMCs by transfecting shILF3. At the same time, the proportion of cells positive for SA β-gal activity was increased in ILF3-silenced VSMCs. Also, ILF3 knockdown significantly decreased cell proliferation, as shown by BrdU incorporation assay. On the contrary, ILF3 overexpression in VSMCs decreased the proportion of cells positive for SA β-gal activity and downregulated the expression of p21 and p16, accompanied by an increased level of CDK4. These results indicated that ILF3 inhibits VSMC senescence induced by chronic Ang II stimulation, which is in keeping with previous reports on human fibroblasts [4]. Additionally, another study reported that TCP80, also known as nuclear factor 90 (NF90), promoted cellular senescence in some tumor cell lines [39]. Therefore, we speculated that the roles of ILF3 in cellular senescence may be cell-type and context-dependent. In this study, when ILF3-overexpressing VSMCs were treated with Ang II, cellular senescence promoted by Ang II was significantly suppressed by ILF3 overexpression. These results suggest that ILF3 overexpression inhibits Ang II-induced VSMC senescence via binding with CDK4 mRNA, which renders CDK4 mRNA stable and thus facilitates its translation. Indeed, our data from *in vitro* RIP experiments and RNA pull-down confirmed that ILF3 was able to bind to 3′ UTR of CDK4 mRNA to strengthen the stability of CDK4 mRNA.

Cellular senescence is traditionally defined as a permanent and irreversible proliferative arrest state of cells. CDK4 and its close homolog CDK6 form heterodimers with D-type cyclins, and CDK4/6-mediated deactivation of the retinoblastoma (RB) tumor suppressor is critical for cell-cycle progression [40]. By binding to the D-type cyclins, CDK4 is activated and in turn phosphorylates RB, increasing the expression of E2F target genes and achieving G1–S transition of the cell cycle. CDK4 overexpression in normal cells can delay cellular senescence in response to inducers [41]. On the contrary, CDK4 inhibitors accelerate cellular senescence [42, 43]. In the present study, we found that circACTA2 competed with CDK4 mRNA for ILF3 binding. Importantly, in Ang II-treated VSMCs, increased circACTA2 facilitated its association with ILF3, thus diminishing CDK4 mRNA stability through reducing interaction of ILF3 with CDK4 mRNA. Targeting circACTA2 and its downstream effectors may serve as potential new strategies against VSMC senescence-associated cardiovascular diseases.

## MATERIALS AND METHODS

### Clinical samples

In this research, the renal arteries were obtained from twenty-four patients of the second hospital of Hebei Medical University (Shijiazhuang, China) from 2017 to 2019, twelve with high blood pressure at least ten years and twelve without. The hypertensive patients lowered their blood pressure with the help of hypotensor. The protocols for this research were supported by the ethical committees. Before donating their tissues, all participants signed informed consents. Half of the vascular samples were fixed overnight in 10% formalin for paraffin embedding. Half of the vascular samples were snap-frozen for further nucleic acid extraction.

### Cell culture and treatment

Human aortic smooth muscle cells (VSMCs) (ScienCell, no. 6110) were cultured in Smooth Muscle Cell Medium containing 2% fetal bovine serum (FBS), fibroblast growth factor-2, hydrocortisone, apo-transferrin, insulin, and insulin-like growth factor-1 (ScienCell, no. 1101). Ang II (100 nmol/L) was used to stimulate VSMCs for 1, 3 or 5 days. VSMCs were serum-starved for 24 h before stimulated by Ang II (R&D Systems, Minneapolis, MN, USA). Human embryonic kidney 293A cells were purchased from ATCC (Manassas, VA, USA) and were cultured in high glucose DMEM supplemented with 10% FBS.

### LC–MS/MS analysis (RAP, pulldown and co-IP MS)

Filter-aided sample preparation (FASP) method was used to handle protein samples. Proteins were hydrolyzed to peptides by trypsin digestion, and peptide solution was prepared for further LC–MS/MS analysis. The peptide was separated by a Nano-HPLC (EASY-nLC1200) which has a reversed-phase column (100 μm, ID × 15 cm, Reprosil-Pur 120 C18-AQ, 1.9 um, Dr. Math). Samples were separated at a 300 nl/min flow rate with the following gradient: 0–40 min, 5–30% B; 40–54 min, 30–50% B; 54–55 min, 50–100% B; 55–60 min, 100% B. Mobile phases A and B were H_2_O-FA and ACN-H_2_O-FA, respectively. Q-Exactive mass spectrometry (ThermoFinnigan) was used for peptide scanning. MaxQuant (Version 1.5.6.0) was used for processing raw MS files. The human protein sequence database (Uniprot_HUMAN_2016_09) was downloaded from UNIPROT.

### Plasmid constructs

The expression plasmid of circACTA2 was constructed by inserting human circACTA2 cDNA into the circpcDNA3.1 vector. The expression plasmid of ILF3 was constructed by inserting human ILF3 full-length cDNA into the pcDNA3.1 vector.

### Senescence-associated β-galactosidase activity assay

Senescent Cells Staining Kit (Cell Signaling Technology) was used as previously described [44] to assay cellular SA-β-gal activity. Briefly, we used pre-cold PBS to wash VSMCs for three times and fixed cells for 15 min in 4% paraformaldehyde at room temperature. When fixation was finished, we washed cells with PBS three times and then incubated cells in SA-β-gal activity solution (pH 6.0) at 37°C for 24 h. Then the enzymatic reaction was stopped by another PBS washing. Images were obtained by light microscope and taken from random fields in each sample. Image-Pro Plus 6.0 software (Bethesda, MD, USA) was used to determine the ratio of positive cells to total cells.

### Western blot analysis

Proteins from cultured cells and artery tissues were prepared with RIPA buffer (Solarbio, China). Western blot protocols were as described before [20]. Antibodies used in this work were as followings: anti-ILF3 (1:1000, ab126742), anti-p21 (1:500, sc-348), anti-p16 (1:500, sc-393009), anti-CDK4 (1:500, sc-130617) or anti-β-actin (1:1000, sc-47778). All experiments were replicated three times.

### Co-immunoprecipitation assay

Co-immunoprecipitation was carried out as previously described [20]. In short, after immunoprecipitating the cellular lysates with anti-ILF3 for 1 h, protein A-agarose was incubated together overnight. Then we collected the protein A-agarose-antigen-antibody complexes and washed them for 5 times with 1 ml immunoprecipitation-HAT buffer (50 mM Tris-HCl, pH 8.0, 150 mM NaCl, 5 mM EDTA, 0.5% NP-40, and 0.1 mM PMSF). Proteins bounded to ILF3 were resolved by SDS-PAGE, detected by Western blotting with anti-ILF3 antibody.

### Isolation of RNA and PCR

QIAzolLysis Reagent (Catalog no.79306) was used to lyse the cultured cells. According to the manufacturer's instructions (miRNeasy Mini Kit; Catalog no.217004), we extracted total RNA from the lysate. A Nanodrop 2000 (Thermo) was used to determine the quality of the RNA. For RNase R treatment: we incubated 5 μg of total RNA and 20 U/μl of RNase R (Epicentre Technologies, Madison, WI, USA) together for 20 min, then a RNeasyMinElute cleaning Kit (Qiagen) was used to purify the resulting RNA. For miRNA: a miRNA Detection Kit from Genepharma (Shanghai, China) was used to perform reverse transcription and qRT-PCR, U6 as internal controls. For large mRNA: an M-MLV First Strand Kit (Life Technologies) was used to synthesize cDNA, random hexamer primers from the Kit were used. Platinum SYBR Green qPCR Super Mix UDG Kit (Invitrogen) was used to perform qRT-PCR of mRNAs or circRNAs. An ABI 7500 FAST system (Life Technologies) was used to carry out real-time PCR experiments. As previously described [45], relative amount of transcripts was normalized with GAPDH and calculated using the 2^−ΔΔCt^ formula. RT-PCR of mRNAs or circRNAs: KOD Xtreme™ HotStart Polymerase Kit (71975-3, Novagen) was used to amplify 1:5 diluted cDNA or gDNA in a 25 μl PCR reaction (22–37 cycles, depending on the template). QIA quick PCR purification kit (Qiagen) was used to purify the PCR products. GENEWIZ services performed direct PCR product Sanger sequencing. Supplementary Table 1 summarizes the primer sequences.

### Short hairpin RNA (shRNA) expression vector construction and transfection

Oligonucleotides were designed specific for circACTA2 or ILF3 mRNA, after annealing the formed double-stranded DNAs were ligated to plasmid pcDNA3.1. The expression vectors of pcDNA3.1-shcircACTA2 or pcDNA3.1-shILF3 were identified by enzyme digestion and sequence analysis and then transfected into VSMCs using Lipofectamine 2000 following the manufacturer's instructions. After twenty-hours of transfection, VSMCs were treated with Ang II. Cells were then harvested and lysed for western blotting or PCR.

### Biotin pull-down of RNA

Biotin pull-down was performed as previously described [45]. In short, we cross-linked VSMCs with 1% formaldehyde in PBS for 10 min, then 0.125 M glycine was used to stop the reaction. Lysis buffer containing complete protease inhibitor and RNase inhibitor was used to resuspend the cells, and sonication was performed later. Two times volume of hybridization buffer and 100 pmol biotin probes (sequences in Supplementary Table 1) were added to the cellular lysate. Yeast tRNA and BSA were used to block Streptavidin Dynabeads (Life Technologies) for 2 h. 100 μl washed/blocked Dynabeads was added per 100 pmol of biotin probes, and the whole mix was then rotated for 30 min at 37°C. Magnets (Life Technologies) were used to capture the beads and wash buffer was used to wash the beads for five times. Beads were then subjected to RNA extraction with elution buffer.

### Fluorescence *in situ* hybridization

4% paraformaldehyde was used to fix cells cultured on coverglass. Paraffin cross-sections (4 μm thick) from renal arteries were deparaffinized and rehydrated for hybridization. Under the instructions of miRCURY LNA™ microRNA ISH Optimization Kit (Exiqon), specific probes of circACTA2 were used to perform *in situ* hybridization. These fluorescence-labeled probes (Supplementary Table 1) were incubated in hybridization buffer (Exiqon) at 55°C in a thermoblock (Labnet) to perform hybridization. Then we washed the glasses with SSC buffer and stained the nuclei with DAPI (157574, MB biomedical). A Leica microscope (Leica DM6000B, Switzerland) was used to acquire images and a software of LAS V.4.4 (Leica) was used to digitize them.

### RNA immunoprecipitation (RIP) assay

RIP was carried out as previously described [45]. In short, VSMCs were harvested and NETN buffer was used to lyse cells. The Dynabeads™ Protein G Immunoprecipitation Kit (10007D, Thermo Fisher), anti-ILF3 antibody or IgG were used to conduct RIP experiments according to the manufacturer's instructions. After washing the beads with NETN buffer for three times, RNA Purification Kit (RNAeasy Mini Elute kit, QIAGEN) was used to extract RNA according to the manufacturer's protocol. NanoDrop 2000 (Thermo-Fisher) was used to quantify the RNA fraction isolated by RIP. Primers used for RT-qPCR were as followings: Cyclin E1-UTR-F:CGTGCGTTTGCTTTTACAGA, Cyclin E1-UTR-R: AGCACCTTCCATAGCAGCAT; CDK4-UTR-F: GGGCCGAGAGGACAGAATGG, CDK4-UTR-R: GCTGTTCTAATCACCAGGGTAGGCC; Cyclin D1-UTR-F: AGCGCTGTTTTTGTTGTGTG, Cyclin D1-UTR-R: TCATCCTGGCAATGTGAGAA; GAPDH-F: ATGAATGGGCAGCCGTTAGG, GAPDH-R: TGGAATTTGCCATGGGTGGA.

### GST pull-down assay

Glutathione S-transferase (GST) and GST-ILF3 fusion protein were produced by BL21 Escherichia coli under induction of isopropylthio-β-galactoside at 28°C. The purification of the proteins was performed by affinity absorption with glutathione-Sepharose 4B beads (Amersham Biosciences, Uppsala, Sweden). Total cell lysates, the recombinant GST and GST-ILF3 proteins on the glutathione beads were incubated together overnight, followed by extensive washing. qRT-PCR was performed to quantify the RNAs on the beads.

### Statistical analysis of experimental data

All of the data are presented as the means ± SEM. Analysis of variance followed by a Student's *t*-test was used to assess differences between two groups. ANOVA or repeated ANOVA followed by Tukey’s posthoc test were used for multiple comparisons or repeated measurements. When a value of *P* < 0.05 was presented, we considered that it was statistically significant. And we denoted with 1, 2, or 3 asterisks when the value was lower than 0.05, 0.01, or 0.001, respectively. Graphpad Prism 5 software (GraphPad Software, San Diego, CA, USA) was used to perform statistical analysis.

## Supplementary Materials

Supplementary Figures

Supplementary Table 1
