# Correction for: circACTA2 mediates Ang II-induced VSMC senescence by modulation of the interaction of ILF3 with CDK4 mRNA

**DOI:** 10.18632/aging.204274

**Published:** 2022-09-12

**Authors:** Ying Ma, Bin Zheng, Xin-Hua Zhang, Zi-Yuan Nie, Jing Yu, Hong Zhang, Dan-Dan Wang, Bei Shi, Yang Bai, Zhan Yang, Jin-Kun Wen

**Affiliations:** 1 Department of Biochemistry and Molecular Biology, Key Laboratory of Neural and Vascular Biology, Ministry of Education, Hebei Medical University, Shijiazhuang, 050017, China; 2 Department of Urology, The Second Hospital of Hebei Medical University, Shijiazhuang, 050000, China; 3 Department of Biochemistry and Molecular Biology, Binzhou Medical University, Yantai, 264003, China

**This article has been corrected:** The authors requested replacement of **Figure 3** and **Figure 5**, in which the images for **Figure 3B** (SA-β-gal staining in VSMCs transfected with shILF3, the right subpanel) and **Figure 5D** (SA-β-gal staining in VSMCs transfected with Vector + circACTA2, the upper right subpanel) were incorrectly placed during the assembly of the figures, resulting in accidental duplication of the images of SA-β-gal staining in Figure 3B, Figure 3I (SA-β-gal staining on VSMCs treated with Vector + AngII, the upper right subpanel*)* and Figure 5D. The authors corrected these figures by using the correct images from the original sets of experiments. These corrections do not affect the article's conclusions. The authors would like to apologize for any inconvenience caused.

New **Figures 3** and **5** are presented below.

**Figure 3:**
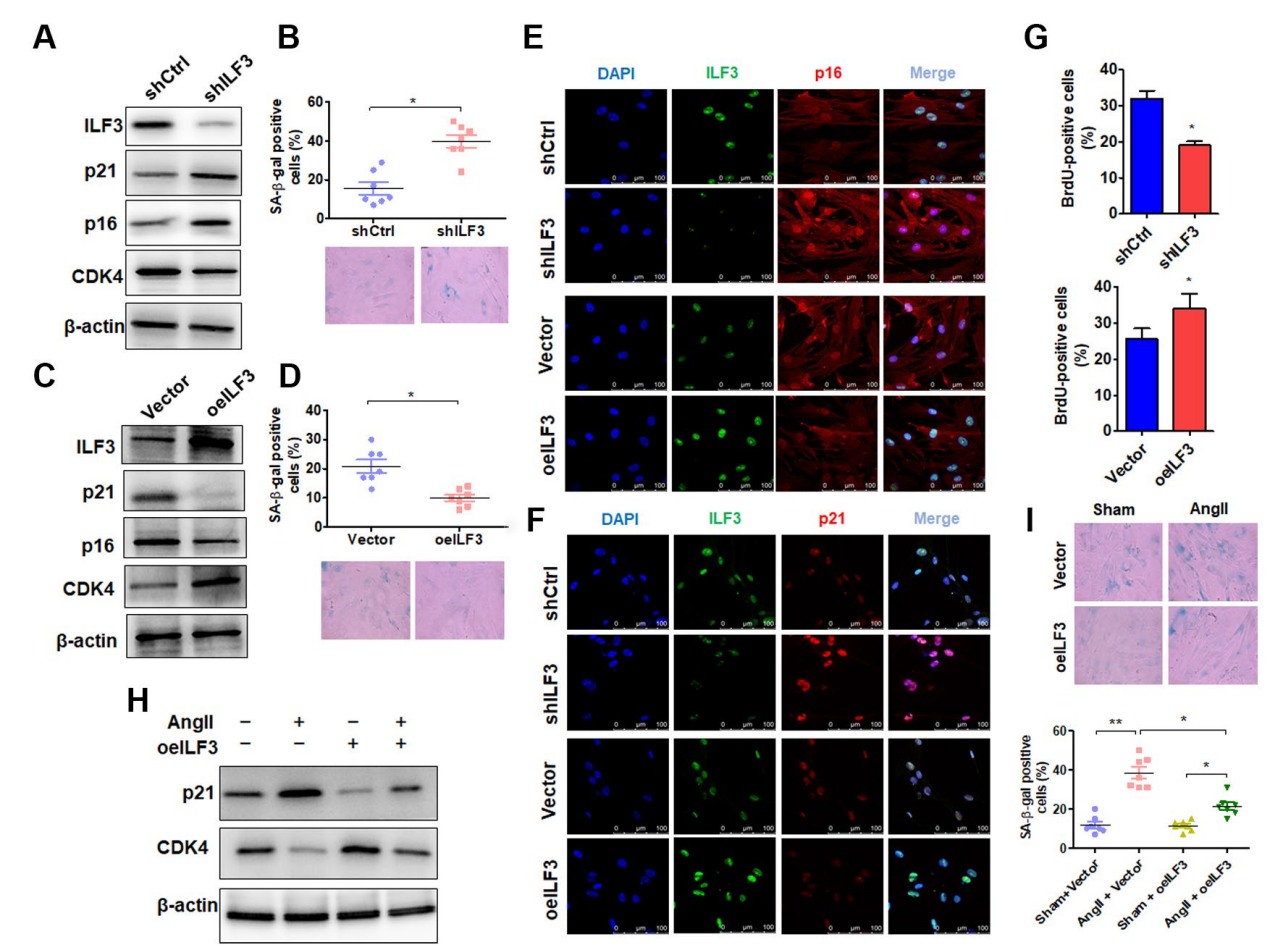
**ILF3 overexpression suppresses Ang II-induced VSMC senescence.** (**A**) Western blot detection of ILF3, p21, p16 and CDK4 expression in VSMCs transfected with shCtrl or shILF3. (**B**) SA-β-gal activity in VSMCs transfected with shCtrl or shILF3. The percentage of SA-β-gal positive cells (above) and representative pictures (below) are shown. Magnification × 400. *P < 0.05 vs. shCtrl. (**C**) Western blot detection of ILF3, p21, p16 and CDK4 expression in VSMCs transfected with empty vector or ILF3-expressing vector (oeILF3). (**D**) SA-β-gal activity in VSMCs transfected with empty vector or oeILF3. The percentage of SA-β-gal positive cells (above) and representative pictures (below) are shown. Magnification ×400. *P < 0.05 vs. empty vector. (**E**) and (**F**) VSMCs were transfected with shILF3 or oeILF3 and their corresponding controls, and the expression of ILF3, p16 and p21 was examined by immunofluorescence staining. Green, red, and blue staining indicates ILF3, p16 (E), p21 (F) and the nuclei, respectively. Scale bar = 100 μm. (**G**) VSMCs were transfected as in (E), and cell proliferation was estimated by BrdU incorporation test. Graph presents means ± SD from at least three independent experiments. *P < 0.05 vs. their corresponding control. (**H**) Western blot detection of p21 and CDK4 expression in VSMCs transfected with empty vector or oeILF3 followed by treatment with or without Ang II. (**I**) SA-β-gal activity in VSMCs transfected with empty vector or oeILF3 followed by treatment with or without Ang II. The percentage of SA-β-gal positive cells (bottom) and representative pictures (top) are shown. Magnification × 400. *P < 0.05, **P < 0.01 vs. their corresponding control.

**Figure 5:**
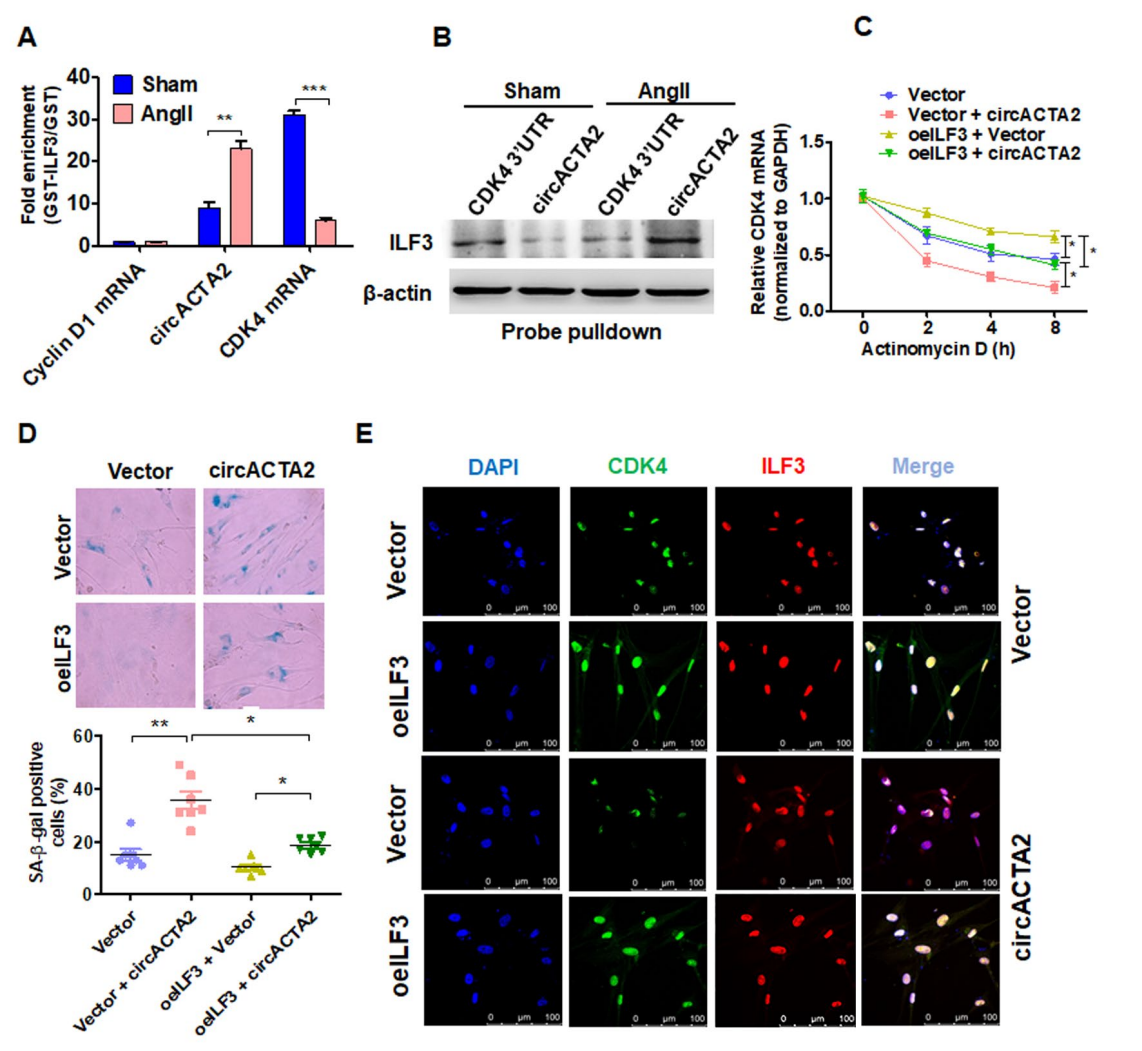
**circACTA2 promotes VSMC senescence by competing with CDK4 mRNA to bind to ILF3.** (**A**) circACTA2, cyclin D1 and CDK4 mRNAs were pulled down with recombinant GST-ILF3 from the lysates of VSMCs treated with or without Ang II for 3 days. The cyclin D1 and CDK4 mRNA as well as circACTA2 on the beads were subjected to qRT-PCR detection. **P < 0.01, ***P < 0.001 vs. vehicle control. (**B**) The lysates of VSMCs treated with or without Ang II were pulled down with CDK4 3′ UTR or circACTA2 probe, and ILF3 in the precipitates was detected by Western blot analysis. (**C**) VSMCs were transfected with circACTA2 and ILF3-expressing vector (oeILF3) either alone or together. Then cells were exposed to actinomycin D for 0, 2, 4, and 8 h. CDK4 mRNA level was detected by qRT-PCR. *P < 0.05 vs. their corresponding control. (**D**) SA-β-gal activity in VSMCs transfected as in (C). The percentage of SA-β-gal positive cells (bottom) and representative pictures (top) are shown. Magnification × 400. *P < 0.05, **P < 0.01 vs. their corresponding control. (**E**) VSMCs were transfected as in (C), and the expression of CDK4 and ILF3 was examined by immunofluorescence staining. Green, red, and blue staining indicates CDK4, ILF3, and the nuclei, respectively. Scale bar = 100 μm.

