# Supplementary material for: circACTA2 mediates Ang II-induced VSMC senescence by modulation of the interaction of ILF3 with CDK4 mRNA
Source: Aging (Albany NY). 2021 Apr 22;13(8):11610–28. doi: 10.18632/aging.202855 (PMC8109074; doi:10.18632/aging.202855)
Supplement: Supplementary Figures [file aging-13-202855-s001.pdf]

SUPPLEMENTARY FIGURES

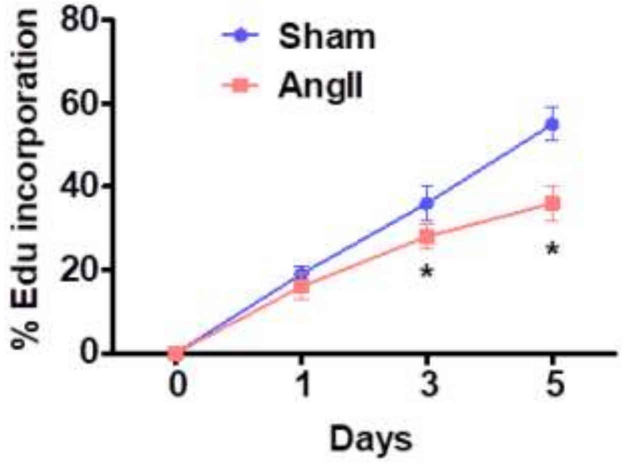

Supplementary Figure 1. **EdU incorporation assay in VSMCs treated with Sham (vehicle) or Ang II for different times.** $^{*}P$ < 0.05 vs. Sham; n = 5.

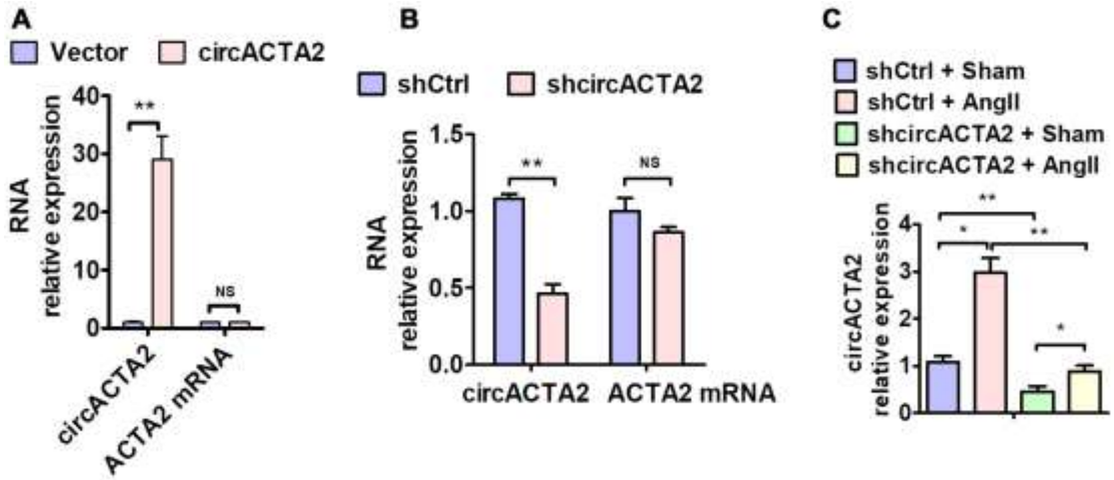

Supplementary Figure 2. (**A**) RT-qPCR detected circACTA2 and ACTA2 mRNA expression in VSMCs transfected with empty vector or circACTA2 expression vectors. (**B**) VSMCs were transfected with shCtrl or shcircACTA2 vector, and circACTA2 and ACTA2 mRNA expression was detected by RT-qPCR. (**C**) RT-qPCR detected circACTA2 expression in VSMCs treated as indicated. $^{*}P$ < 0.05, $^{**}P$ < 0.01 vs. their corresponding control.

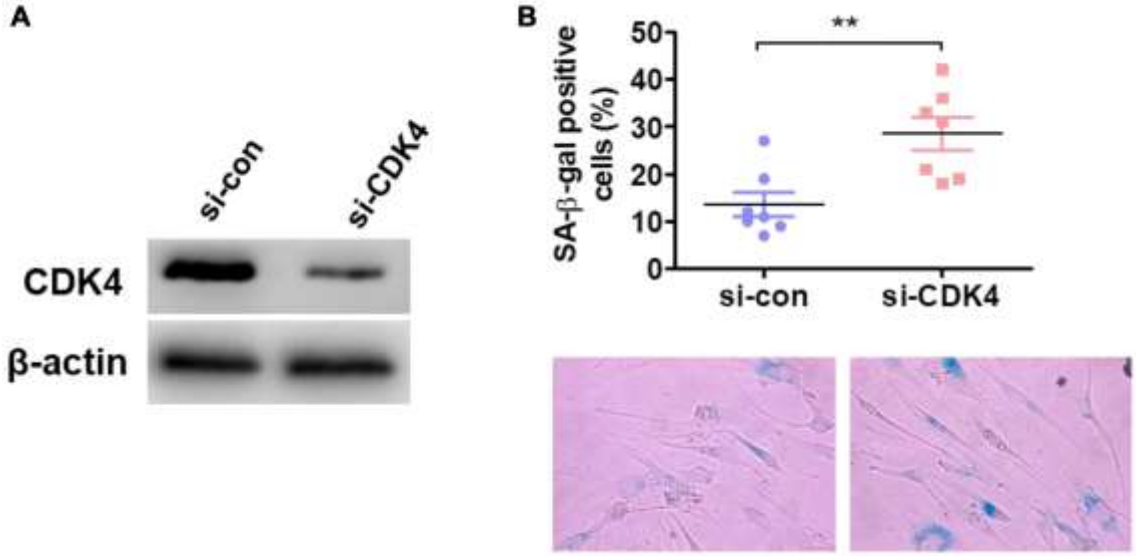

Supplementary Figure 3. Knockdown of CDK4 induces senescence of VSMCs. (**A**) Western blot detected the expression of CDK4 in VSMCs transfected with si-CDK4 or si-con for 48 h. (**B**) SA-β-gal activity in VSMCs transfected with si-con or si-CDK4. The percentage of SA-β-gal positive cells (above) and representative pictures (below) are shown. Magnification × 400. $^{**}P < 0.01$ vs. vehicle control.

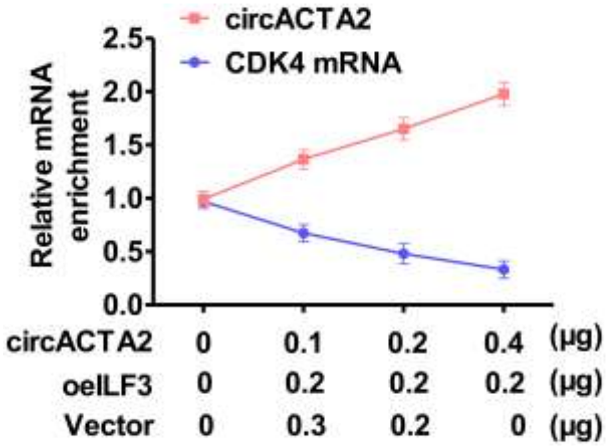

Supplementary Figure 4. **RIP-PCR detected circACTA2 competition with CDK4 mRNA for binding with ILF3.** VSMCs were co-transfected with increasing amounts of circACTA2 expression plasmids and a constant amount of ILF3-expressing vector, and then anti-ILF3 antibody was used to immunoprecipitate RNAs binding to ILF3. PCR detected the enrichment of circACTA2 and CDK4 mRNA in the anti-ILF3 immunoprecipitates.
