# Supplementary material for: circACTA2 mediates Ang II-induced VSMC senescence by modulation of the interaction of ILF3 with CDK4 mRNA
Source: Aging (Albany NY). 2021 Apr 22;13(8):11610–28. doi: 10.18632/aging.202855 (PMC8109074; doi:10.18632/aging.202855)
Supplement: Supplementary Table 1 [file aging-13-202855-s002.pdf]

SUPPLEMENTARY TABLE

Supplementary Table 1. Oligos used in the study.

Name	Sequences 5' to 3'
circACTA2-F	GGCAACTCGTAACTCTTCTCAAGGG
circACTA2-R	CTCTGTCTGGATCGGTGGCTCC
circACTA2-F	GGAATGATTTGGAAAAGAACTGAAGGC
circACTA2-R	CTCTGTCTGGATCGGTGGCTCC
GAPDH-F	AAAGCCTGCCGGTGACTAAC
GAPDH-R	AGGAAAAGCATCACCCGGAG
CDK4 mRNA-F	GTGTATGGGGCCGTAGGAAC
CDK4 mRNA-R	CAGTCGCCTCAGTAAAGCCA
cyclin E1-F	CCATCATGCCGAGGGAGC
cyclin E1-R	TTTGCCCAGCTCAGTACAGG
p16-F	CCGAATAGTTACGGTCGGAGG
p16-R	AATCGGGGATGTCTGAGGGA
p21-F	AGTCAGTTCCTTGTGGAGCC
p21-R	CATTAGCGCATCACAGTCGC
CDK4-probe1	CATGTCCACAGGTGTTGCATATGTGGACTG
CDK4-probe2	CCATGGCAGCCACTCCATTGCTCACTCCGG
cyclin E1-probe1	CTCCCTGTGAAGTTTATAGACTTCACACAC
cyclin E1-probe2	CTCTGCAATCTGTATAAAGATTTGCTGGGG
cyclin D1-probe1	GTCACACTTGATCACTCTGGAGAGGAAGCG
cyclin D1-probe2	CCTCCACTGGATGGTTTGTCACTGGATGGT
